# Ten simple rules for a department chair

**DOI:** 10.1371/journal.pcbi.1014934

**Published:** 2026-11-06

**Authors:** Lubomir Tomaska

**Affiliations:** Faculty of Natural Sciences, Department of Genetics, Comenius University Bratislava, Bratislava, Slovakia; Carnegie Mellon University, UNITED STATES OF AMERICA

## Abstract

Department chairs occupy a pivotal position in the university. Teaching, research, graduate education, and much of the academic community's professional and administrative activity are organized at the departmental level. Yet, despite the importance of the position and the considerable challenges it entails, many scientists assume the role with little or no formal preparation for academic leadership. This paper is intended as a practical primer rather than a comprehensive guide to academic leadership. The proposed rules provide a framework that prospective and current department chairs can adapt to their own institutional circumstances, experience, and leadership style.

## Introduction

Entering academia may be accompanied by many dreams: discovering something fundamental, developing a treatment for a previously untreatable disease, becoming a leader in one's field or a master of a particular technique, becoming an excellent teacher and mentor, or simply enjoying work that is both interesting and meaningful. Although I have no empirical evidence, I suspect that becoming a department chair rarely appears on the list of aspirations of a novice scholar.

Yet, as argued by Walt Gmelch in his insightful series of texts on the subject (*e.g.*, [1]), department chairs occupy the most important position in the university hierarchy. This is because teaching, research, graduate programs, and service to the professional community all take place at the departmental level. Indeed, according to some estimates, 80% of university decisions are made at the department level [2]. Deans are, of course, also essential, but their effectiveness depends to a large extent on the effectiveness of their department chairs.

Importantly, the position of department chairs is highly distinctive. They carry a full load of administrative and managerial responsibilities (often without a substantial financial compensation) while usually retaining their other professional roles as teachers and researchers. Indeed, 96% of department chairs perceive themselves primarily as faculty members, whereas only 4% identify as administrators [1]. Yet, balancing these competing demands can be challenging, as time devoted to administration inevitably comes at the expense of time available for research, teaching, and creative scholarly work.

Given the importance of the position and the challenges it entails, only a small fraction of department chairs (around 3% in the US [3]) receive formal leadership training. Considering the fact that expertise typically requires extensive (several years) deliberate practice (*e.g*., [4]), a department chair who relies solely on experience, without formal preparation, often becomes an expert in managing the department just as his or her term is about to expire.

Despite the challenges associated with the position, my personal experience leads me to agree with the statement that “Serving as a department chair can be rewarding […] through working with students, faculty, and administration” [5]. In hindsight, however, I believe that had I entered the office better prepared, those rewards would have become apparent much sooner. The purpose of this essay is to offer those considering this stage of their professional career some advice on how to increase their chances of making the most of the position. This text is not intended to provide a comprehensive guide for prospective department chairs, as an extensive literature on the subject already exists (*e.g.,* [1–8]; there is even a dedicated journal *The Department Chair*, https://onlinelibrary.wiley.com/journal/19364393). Rather, the rules presented below represent a synthesis of insights from the literature, blended with my own experience. I also recognize that experienced academic leaders may have different perspectives on some of the proposed rules, or may suggest additional ones. If the article stimulates discussion among department chairs, faculty members, and prospective academic leaders about how departments can be managed more effectively and collegially, it will have achieved its purpose.

## Rule 1: Enter the office with meaningful motivations and well prepared

The motivations for becoming a department chair can be quite diverse and may include the desire to (1) improve the department; (2) pursue personal and professional development; (3) gain greater control over one's work environment; (4) respond to the persuasion of others (*e.g*., the dean or colleagues); (5) take advantage of an opportunity to facilitate a transfer to another institution; or (6) accept the position out of a sense of duty or because nobody else is willing to do it [5,6]. Interestingly, those chairs that are motivated by intrinsic (examples 1–3 above) rather than extrinsic (examples 4–6) reasons are three-times more likely to continue in the position in the second term [1]. Although there are no data on the correlation of the motifs and performance of department chairs, they seem to affect their persistence, which may be important for implementation of department changes that require a longer time extending one term.

Regardless of the motivation, one should have a clear plan of what to achieve in the short and long term. Such a plan is often required as part of the application process and can serve as the starting point for what may be called the preparatory phase of the job. The ultimate measure of your department’s success is the quality of education it provides to its students. Therefore, whatever your particular plans and priorities, keep this goal at their core: to provide your students with the best education possible.

Once the application is successful and the position is accepted, the preparatory phase continues. It is highly advisable to go through a transient period when the previous chair is still in office. Even with a detailed plan, a new chair needs to become familiar with many administrative and technical nuances. Ideally, the institution's administrative infrastructure should absorb as much of the bureaucratic burden as possible, allowing department chairs to focus on strategic leadership and academic matters. However, one should be prepared for a less optimistic scenario and obtain a realistic picture of the administrative responsibilities associated with the position as early as possible. The old chair can be an invaluable chaperone in the administrative maze thus making the transition much more effective.

In the ideal scenario, the preparatory phase is used to get a formal training in managerial and/or leadership skills. Even if you are a principal investigator and thus have an experience with leading a group of researchers and students, you are still a specialist, while department chairs must be generalists dealing with a much wider repertoire of problems. One can argue that you can learn these generalist skills empirically, but the mistakes you make during such self-learning will negatively affect lives of your colleagues and students, so it is much more desirable to get a “vaccination” at least against those preventable errors in a form of dedicated courses or workshops. If your university does not provide such training, seek them by yourself and ask the dean to refund the costs with the rationale highlighted above.

## Rule 2: Build a team, not a collection of superstars

Your plans can be both ambitious and well-drafted, but for their implementation you will need help from the members of your department. They will be partly inherited and partly recruited. It will be to a large extent up to you to blend an optimal mix. How to proceed?

It might seem that the answer to this question is trivial: one should try to identify the best of the best and assemble a winning team. Wang and Barabási [9] use the following story illustrating that it is not that simple. In a well-known study, William Muir, a professor of zoology at Purdue University, tested this hypothesis—on chickens. He placed groups of chickens with two different characteristics into cages. The first category consisted of chickens that were housed in cages with the highest average productivity. The second category consisted of superstar egg-laying hens. He bred individuals within these groups for six generations while simultaneously measuring how the average egg production per cage changed—in other words, he measured team performance. He found that while the first category of chickens gradually improved their performance as a team, the second category underwent a genocide. The average number of chickens per cage dropped from nine to three stressed individuals who, understandably, had neither the strength nor the motivation to lay eggs [10].

This situation is by no means limited to poor birds in cages; it also applies to human teams. Examples can be found particularly in team sports, where the so-called too-much-talent effect is clearly evident. But it applies to academia as well. In the 1980s, a Department of English was established at Duke University in North Carolina, whose members were all superstars—something like a Who’s Who in English—recruited from across the United States. Immediately afterward, the number of applicants, grants, and publications increased, as did the superlatives used by accreditation committees to describe the department. Until, after a few years, the entire collective dissolved like sugar in water due to hypertrophic non-complementary egos [9].

Science and teaching are collective activities that require the right balance of talent, specific individual skills, healthy self-confidence, but also humility and the ability to cooperate. Teams mixed in this way exhibit a much higher degree of collective intelligence than teams composed exclusively of superstars eager for individual dominance.

## Rule 3: Use “communal brain” for your decisions

As department chair, you (and only you) will bear ultimate responsibility for decisions affecting departmental matters. Your individual responsibility, however, does not mean that the decisions must be reached solely by you. In his interview for the podcast *Night Science,* the biochemist and protein designer David Baker described his concept of a communal brain: “Really for the lab, each researcher, including me, is like a neuron in the communal brain, and everything we do is sort of trying to maximize connectivity and that's where I think real scientific creativity comes from. […] Not the productivity, but the number of really exciting new ideas and the number of connections in the communal brain that scales. Now it scales provided that all the neurons stay connected” [11]. Although Baker’s metaphor is described for a research group, it is also valid for the university department dealing with questions that require creativity and out-of-the-box thinking such as a design of a new curriculum for a study program, implementation of AI to teaching, or optimization of the departmental infrastructure. If you put together a good mix of people with complementary strengths (Rule 2), you can expect that they constitute a communal brain producing valuable and emergent solutions. This approach is consistent with the concept of collective intelligence [12], which describes a group’s general capacity to perform effectively across different types of tasks. Interestingly, collective intelligence appears to depend less on the individual intelligence of group members than on how they interact with one another. Groups tend to perform better when their members demonstrate greater social sensitivity, participate relatively equally in discussions, and include a higher proportion of women. Thus, the effectiveness of a team may be determined more by its composition and patterns of interaction than by the abilities of its individual members [9].

Just as in case of an actual brain, not all neurons must fire all the time. On the contrary, they should be activated only when needed to solve a problem worthy their attention. Filter out ballast. More specifically, when you are dealing with trivial administrative tasks, do not bother your colleagues. On the other hand, when you have to reach a strategic decision, constitute working groups consisting of those who have the best potential to find the best solution. The platform for such discussions can be, depending on the nature of the problem, either a formal meeting (Rule 6), or an informal departmental event (Rule 7).

## Rule 4: Cultivate sound relationships with the university officials

In many instances, your decisions will be limited by the formal institutional rules and/or the willingness of the university officials to help you to achieve your goals. Therefore, your relationships with the school administrators will, to a large extent, define a range of possibilities available to you to solve a particular problem or to realize an intended task. Therefore, before you start to take practical measures, draft a plan, discuss it with your colleagues at the department and then introduce it to the official to explain your intentions. Be ready to make some adjustments and possibly compromises as not all your ideas will meet with understanding. In many cases this reluctance is not caused by the official’s arrogance or unwillingness to help, but by the formal/legal constraints. Constructive negotiations very often lead to acceptable solutions, sometimes even better than the original plan.

At the same time, department chairs should not view themselves merely as agents of administrative decisions. They also have a responsibility to represent the legitimate interests of faculty, staff, and students, and at times this may require questioning, challenging, or opposing proposals originating from higher levels of the university hierarchy. Such advocacy is most effective when it is evidence-based, communicated professionally, and clearly motivated by the long-term interests of the department and institution. However, be prepared that the interactions with the officials may sometimes yield a sensitive situation with no clear solution. For example, your dean may offer you a large amount of money, additional space, or some other substantial benefit for your department, while expecting you in return to do something that goes against your principles—for example, to hire a person who does not meet your standards or to write a positive review of something or someone you do not consider worthy. Refusing the offer may mean losing a rare opportunity to develop your department, while accepting it may compromise your personal integrity. Although I admit that such a situation may look easier on paper than it is in reality, my advice is to stick to your principles and make your position clear to the official who made the offer. It is also useful to discuss the situation with your colleagues. Even if they do not agree with your decision, they should be familiar with your motivation and know that you made this choice for principled reasons. Although you may thereby deprive your department of a financial or infrastructural boost, this course of action benefits not only your personal credibility but also the reputation of your department.

## Rule 5: Make sure that your colleagues are informed about the departmental matters

To help the members of the department to be effective scientists and good teachers and mentors, you should try your best to minimize their administrative load; it is you and your office that are responsible for dealing with the bureaucracy associated with running the department. On the other hand, all members should have access to the information related to the department. Some may be just posted on the dedicated website platform, others may be communicated via chat/e-mail, or discussed at the regular departmental meetings (Rule 6). If there is a particular task that must be performed, all should know the aims of the task, person(s) responsible for fulfilling it and the deadline. If the aim is not reached, you should provide an explanation; if it was fulfilled, you should acknowledge it. The information flow should be bi-directional: ask your colleagues that if they encountered an information that might be relevant for the department, they should share it with others. Do not allow fragmenting the department into the groups that have a privileged access to some kind of information; this promotes conspiracies and will make your job very difficult. On the other hand, strictly personal information that your colleagues decide to communicate with you (such as health issues, family problems, etc.) should never be shared with others; otherwise you not only lose their trust, but also may eventually cause harm to the corresponding person.

## Rule 6: Make the best out of the departmental meetings

Departmental meetings are often seen as one of the few—sometimes the only—opportunities to bring all members of a department together in the same room. This is certainly an important benefit, but departmental meetings should do better than being a formal routine. They should be “sense-learning events, [where] we learn how our organization is functioning, clarify our roles and expectations, and receive clues about our standing” [13].

Allison M. Vaillancourt uses this quote as a starting point for her condensed and highly practical guide, *How to Run a Good Meeting* [14]. In preparing for a meeting, she suggests considering several key questions: (1) What I wish to accomplish with this meeting (*e.g*., sharing/collecting information, coordination of activities, plotting strategies, generating ideas, learning something). To phrase the question differently: Is this meeting really necessary or can I achieve the intended aims by some less time-consuming strategy; (2) Whom should I invite (small meetings are usually more effective, however, some meetings will have to be attended by all members)); (3) How will the meeting be structured (where it will be held, how long it will take, how many topics will be discussed, who will introduce the particular topic); (4) How will I prevent unproductive discussion, inactivity, resistance to deal with a problem; (5) How will I catalyze reaching a consensus and a decision; (6) How can I make the next meeting better than the previous one. These questions may seem obvious, but considering them before every departmental meeting can help the chair transform a formal administrative obligation into a genuinely useful opportunity for communication, coordination, and collective decision-making.

Some departments organize meetings on the regular basis (*e.g*., once per month on the same day/time). This certainly has some practical benefits, as many topics surface recurrently during the year. However, you should be aware that this periodicity may cause that the meetings are held “for historical rather than practical reasons” and become “little more than memorials to dead problems” [15]. If you prefer to organize regular departmental meetings, make sure that you address the same questions as for impromptu meetings and make them mutually useful for all attendees.

Regardless of the format and content, you should make the minutes from the meeting and make it available to all members of the department as soon as possible. The document should contain list of relevant information, particular tasks, assignments and deadlines that should be checked during the next meeting.

## Rule 7: Foster a culture of mutual support and team spirit

Good departments are composed of gifted individuals who—generally speaking—excel in the quality of their creativity, research outputs, teaching performance, mentoring capabilities or the combination of the above. It is natural that in some respect they are competitors with egos and ambitions. However, at the same time their affiliation makes them members of the same team and their individual success is to a large extent dependent of the success of the department. One of your essential roles as a department chair is to cultivate an environment promoting collaboration, cooperation, and mutual support. For example, make sure that every individual (or group) success (*e.g*., good publication, award) is acknowledged and possibly celebrated by means of a small party (if the personnel is really a good mix, everybody will be recognized). Promote an open-door culture indicating accessibility of the people to colleagues and students. Informal (non-obligatory) interactions (*e.g*., departmental sport-day, hiking tour, Christmas party, a weekend in a cottage) organized at a reasonable frequency is highly instrumental in the identification of common interests among the members of the department and also for discussing different opinions about both work-related and unrelated matters. The goal is not to make everybody friends, but to know each other better thus making it much easier to identify a colleague that can be helpful in solution of a particular problem in the future. Do not forget to invite to all these events all members of the department, including graduate students, technicians, secretaries as they are essential contributors to the department’s performance. Some events should also be attended by the undergraduate students so they get a chance to meet their teachers outside the classrooms and participate in the discussions that may have a positive impact on their education and future careers.

As in any team, you will encounter disagreement between the members of the department. In an ideal world, differences in opinions on a particular problem are a key for finding an optimal solution and thus dissent should be valued. You should be careful in moderating the discussion such that it remains factual and does not become personal. If the latter occurs, it may result in a conflict that should be resolved in the earliest stages possible. You should get familiar with the techniques aimed at handling conflicts by taking formal courses (Rule 1).

It will happen, more often than you wish, that you make a mistake. You may reach a wrong decision, you forget to do something important resulting in a penalty affecting the department, you may unintentionally mistreat someone. Hiding the mistake or blaming others never works. Acknowledge the error and honestly apologize to all affected individuals. It may feel uncomfortable in a short term, but in a long run it fosters an environment of trust in which people are not afraid to acknowledge their mistakes. In addition, it is a sign of a good management. For example, hospital wards that openly report more mistakes have been shown to be better organized and managed than those with apparently error-free records. In the latter, the absence of reported errors may reflect a fear of punishment rather than better performance [16].

## Rule 8: Lead by example

You cannot expect your colleagues to follow rules that you are unwilling or unable to follow yourself. For example, if you want them to be accessible to others, you cannot work permanently behind closed doors. If you want them to respect your privacy and your right to work-free weekends, you should not send them an email on Sunday asking them to complete a task by Monday morning. If you want them to be interested in each other’s research and teaching experiences, you should periodically ask about their progress in the lab and/or classroom. You can hold them accountable for missing a deadline only if you yourself keep your promises to respond to their requests on time.

On the other hand, you may be a workaholic who devotes 16 hours a day to professional activities, but you cannot expect everyone to keep up with you. Be reasonable in your expectations. If you want others to meet certain requirements, first make sure that you are able to meet them yourself. Then discuss the expectations with your colleagues, listen to their perspectives, and, where appropriate, reach a consensus before implementing new rules.

## Rule 9: Evaluate people based on objective, measurable criteria and provide them with means to improve

Quite frequently, you will have to evaluate the performance of members of the department. For example, you may have an additional budget that can be distributed among colleagues based on their performance. The criteria for evaluation may differ between departments, but they should meet two basic requirements. First, they should be as objective and quantifiable as possible; second, they should be known to all members of the department. The first requirement may sometimes be difficult to meet because academic performance is multidimensional, and some important contributions are difficult to quantify. Chairs should therefore avoid the illusion of objectivity and base their judgments on transparent criteria and multiple sources of evidence [17]. The criteria should also be aligned with the department's commitment to academic excellence. While institutions may legitimately pursue multiple objectives, department chairs should ensure that decisions related to hiring, promotion, and rewards remain grounded in merit and professional achievement.

Given the heterogeneity of the group, you are likely to be personally closer to some colleagues than to others. Clearly defined criteria are therefore essential for avoiding decisions influenced by personal preferences or sympathies and antipathies.

The second requirement—transparency of the criteria—helps prevent rumors and suspicions. Make it clear to your colleagues that they are welcome to ask you to explain the reasons for a particular evaluation and to discuss how their performance compares with the established criteria.

The evaluations will lead to a ranking, where some people will lead and others will lag behind in a particular type of activity. While you should acknowledge the leaders, you should also discuss with the others what can be done for their improvement. Sometimes you may discover that you can help them directly by removing a particular obstacle. Even a trivial change, such as modification of a teaching schedule and/or load, can yield a substantially positive result. In other cases you may find that the problem is more systematic. For example, you may discover that some teachers lag behind in their proficiency to use AI in their classes. In such a case you can promote organization of a formal course for teachers to learn and train this particular skill.

## Rule 10: Don't build monuments to yourself; rather build a legacy of departmental excellence

Consider a chair who succeeds in securing substantial financial resources and develops an ambitious vision for how they could be invested in improving the department. Working hard and enthusiastically, s/he implements a series of highly visible changes: renovated hallways, new rooms, new informal spaces for discussions and relaxation. Places that were previously dull and neglected become attractive and welcoming spaces for students, faculty, and staff. Clearly, such efforts can have a positive and lasting impact. Yet even successful changes may not be met with unanimous enthusiasm if members of the academic community have had little opportunity to contribute to the process. When decisions about major changes are made without consulting those who will live and work with them, the improvements may be perceived as someone else’s vision rather than a collective achievement. The leader may be fulfilling a personal dream, but in doing so may miss an opportunity to turn that vision into a shared one.

As a result, the institution may become more attractive and pleasant to work in, but two important opportunities may have been missed. First, the communal brain (Rule 3) could have contributed ideas and perspectives that would have improved the outcome. Second, and more importantly, participation in decision-making helps cultivate a sense of community, collegiality, and loyalty to the institution. A lasting legacy is therefore not simply what a leader leaves behind, but what they enable the community to build together. A collaborative atmosphere in which everyone receives the support needed to reach their full potential and has a voice in decisions affecting the department is an essential prerequisite for departmental excellence. Cultivating such an atmosphere is much more difficult than building monuments to oneself—but it is a far more valuable and lasting legacy.

## Conclusions

In an interview about his career, the geneticist Gerry Fink mentioned his father’s question judging one’s performance at any task: “Did you leave things better than you found them?” [18].

There are various (though debatable) measures of how to evaluate the quality of a scientist, such as his/her publication record, patent applications, and/or the success rate in obtaining competitive grant awards. It is more difficult to evaluate the quality of teachers; yet even in this case one can look at the student evaluations and their performance in their careers. In case of department chairs, the means of measuring their performance is even more challenging. The questions that should be asked to get an idea about the achievements of a department chair are: (1) How did s/he contribute to the advancement of the research and educational programs?; (2) What investments were made into the people (teachers, students, staff) to mediate their personal and professional growth?; and (3) were the goals achieved with decency? These questions correspond to the three themes emerging from the survey of legacy statements provided by several hundred department chairs in Australia [5].

So when you will be leaving the office and you want to self-evaluate your performance, ask yourself: Did I leave things better than I found them and did I do it with decency? If the honest answers will be “yes”, then your mission was worth the effort.
